# Association between estimated glucose disposal rate and preserved ratio impaired spirometry in adults

**DOI:** 10.3389/fendo.2025.1525573

**Published:** 2025-05-16

**Authors:** Tong Lin, Shaofeng Jin, Xingkai Shen, Shanshan Huang, Haiyan Mao

**Affiliations:** ^1^ Department of Critical Care Medicine, Ningbo Medical Center Lihuili Hospital, Ningbo, China; ^2^ Department of Geriatrics, Ningbo Medical Center Lihuili Hospital, Ningbo, China

**Keywords:** estimated glucose disposal rate, eDGR, insulin resistance, preserved ratio impaired lung function, PRISM, lung function, lung injury

## Abstract

**Background:**

Preserved ratio impaired spirometry (PRISm) is a newly defined phenotype of lung function impairment, characterized by a normal FEV1/FVC ratio alongside an FEV1/0.8 < FEV1 predicted value. Previous studies have linked PRISm to various adverse clinical outcomes, but its association with insulin resistance, as indicated by estimated glucose disposal rate (eGDR), remains underexplored.

**Methods:**

A total of 13,661 participants were included in this analysis after excluding individuals with missing data on PRISm (n = 10,954) and eGDR (n = 5,827). The median eGDR for the overall sample was calculated, and differences in baseline characteristics between the PRISm and non-PRISm groups were assessed. Logistic regression models were employed to analyze the relationship between eGDR and PRISm, adjusting for various confounders. Subgroup analyses were conducted based on gender and age. Additionally, the restricted cubic spline analysis was used to evaluate the non-linear relationship between eGDR and PRISm, and ROC analysis was performed to determine the predictive accuracy of eGDR for identifying PRISm.

**Results:**

Participants in the PRISm group exhibited significantly lower median eGDR values compared to the non-PRISm group (9.92 vs. 12.01 mg/kg/min; *P* < 0.001), indicating greater insulin resistance. The weighted multivariable logistic regression analysis revealed that each unit increase in eGDR was associated with a 15.1% reduction in the odds of PRISm in unadjusted models, and 7.3% in fully adjusted models (OR = 0.927, 95% CI: 0.880–0.976; *P* = 0.005). Subgroup analyses demonstrated a stronger association between eGDR and PRISm in females and individuals over 40 years of age. The restricted cubic spline analysis indicated a significant non-linear relationship, with an optimal eGDR cutoff of 11.423 mg/kg/min identified via ROC analysis (AUC = 0.626), demonstrating modest predictive accuracy.

**Conclusion:**

Our study demonstrates a significant inverse association between estimated glucose disposal rate (eGDR) and preserved ratio impaired spirometry (PRISm) among a diverse population of US adults. Participants with lower eGDR values exhibited a higher prevalence of PRISm, indicating greater insulin resistance and potential metabolic dysfunction. The findings suggest that eGDR may serve as a valuable marker for assessing the risk of PRISm, particularly among women and older adults.

## Introduction

Chronic lung disease affects hundreds of millions of people worldwide and ranks as the third leading cause of death globally, following cardiovascular disease and cancer (1). Common lung diseases, such as asthma, chronic obstructive pulmonary disease (COPD), and bronchiectasis, often lead to significant changes in lung function, particularly resulting in airflow obstruction (2, 3). This obstruction is typically identified through lung function testing conducted after administering a bronchodilator, characterized by a reduced ratio of forced expiratory volume in one second to forced vital capacity (FEV1/FVC). In contrast, non-obstructive lung function abnormalities, commonly referred to as restrictive lung disease, are marked by a symmetric reduction in both FEV1 and FVC (4).

However, preserved ratio impaired lung function (PRISm) is a relatively underexplored lung disease that is characterized by a decrease in FVC while the ratio of forced expiratory volume in one second to forced vital capacity FEV1/FVC remains within the normal range, with the global prevalence of PRISm estimated to be between 6.6% and 17.6% (5). Although PRISm has historically been viewed as a transitional state between normal lung function and COPD, retrospective studies have shown that only approximately 23% of individuals with PRISm progress to COPD (6). Some studies have shown that PRISm is significantly associated with increased risks of mortality, as well as adverse cardiovascular and respiratory outcomes (4), and is linked to a higher prevalence of diabetes, heart disease, and hypertension among individuals with chronic diseases (7, 8). In contrast, PRISm has been independently linked to higher cardiovascular risk and increased mortality (9). It may represent a distinct clinical phenotype with unique pathophysiological and prognostic implications, rather than merely an early stage of obstructive lung disease (10).

Insulin resistance is a condition characterized by a diminished response to insulin, which results in decreased efficiency of glucose uptake and utilization, ultimately leading to metabolic abnormalities and serving as a significant risk factor for various metabolic disorders such as type 2 diabetes, hypertension, dyslipidemia, and obesity (11, 12). Recent research has highlighted the correlation between insulin resistance and pulmonary diseases, including impaired lung function and asthma, indicating that individuals with insulin resistance often experience compromised respiratory health, which suggests that metabolic dysregulation may exert both direct and indirect effects on lung function (13–15).

The concept of estimated glucose disposal rate (eGDR) has emerged as a valuable tool for assessing insulin sensitivity, particularly in individuals with diabetes. eGDR is derived from clinical parameters such as body mass index (BMI) and blood pressure, making it a useful surrogate marker for insulin resistance (16). Compared to other insulin resistance markers such as TyG, TyG-BMI, and METS-IR, recent studies have shown that eGDR has superior predictive ability for adverse cardiometabolic outcomes, including stroke and cardiovascular disease (17, 18). While the relationship between insulin resistance and various metabolic disorders has been extensively studied, research exploring the connection between insulin resistance and PRISm remains relatively scarce. These findings suggest that eGDR may also be a more effective indicator for identifying individuals at risk of PRISm, particularly in populations with metabolic disturbances. Therefore, we utilized the national health and nutrition examination surveys (NHANES) database to explore the association between eGDR and PRISm, aiming to further elucidate their potential link in metabolic health and lung function. We hypothesized that decreased insulin sensitivity, as reflected by lower eGDR, would be negatively associated with lung function and increase the likelihood of PRISm, thereby linking metabolic and respiratory health.

## Methods

### Study and data

**Figure 1:**
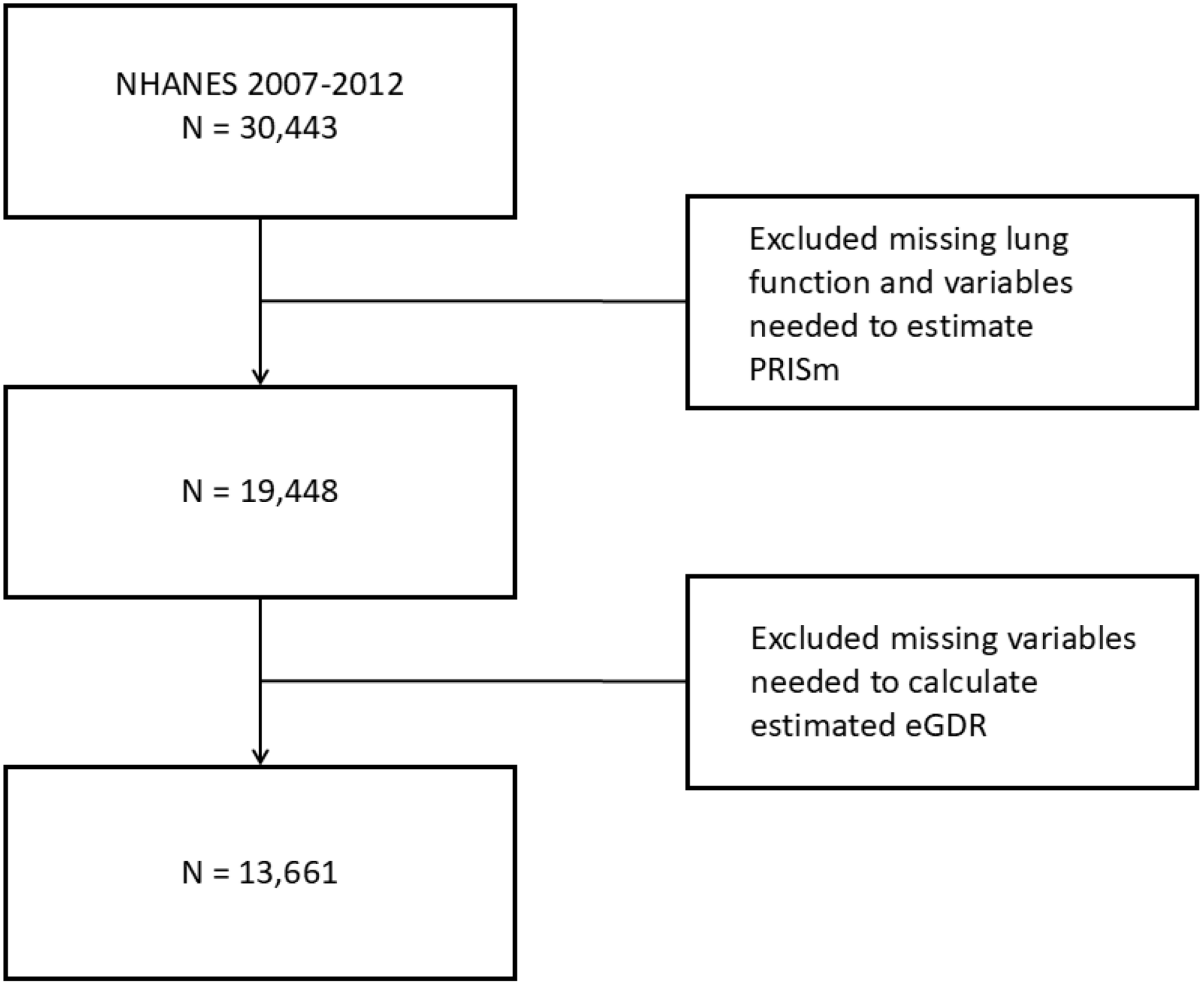
Flowchart depicting the screening process for selecting the study population.

The National Health and Nutrition Examination Survey (NHANES), conducted by the National Center for Health Statistics (NCHS) at the U.S. Centers for Disease Control and Prevention (CDC), is a cross-sectional survey employing a complex, multistage sampling design to gather data representative of the non-institutionalized U.S. population. NHANES operates in two-year cycles, collecting data through in-home interviews and standardized physical examinations. For this study, data from three NHANES cycles (2007–2008, 2009–2010 and 2011–2012) were utilized, based on the availability of lung function measurements. The dataset can be accessed at (https://www.cdc.gov/nchs/nhanes/index.htm). The study population included U.S. adults aged 20–79 who met the criteria for valid spirometry testing. Participants with missing lung function data or essential variables required to estimate predicted forced expiratory volume in one second (FEV1) or to calculate estimated glucose disposal rate (eGDR) were excluded. The participant selection flowchart is illustrated in **Figure 1**.

### Definitions of eGDR and PRISm

The insulin resistance index, estimated glucose disposal rate (eGDR), was calculated using the following equation: eGDR = 21.158 - (0.09 × waist circumference [cm]) - (3.407 × hypertension [yes = 1, no = 0]) - (0.551 × glycated hemoglobin A1c [HbA1c] [%]) (16). Hypertension was defined as (1) systolic blood pressure ≥140 mmHg or diastolic blood pressure ≥90 mmHg, (2) self-reported physician diagnosis of hypertension, or (3) use of antihypertensive medication. Preserved Ratio Impaired Spirometry (PRISm) was defined as a forced expiratory volume in one second/forced vital capacity ratio (FEV1/FVC) ≥0.7 with an abnormal spirometry result (FEV1 <80% of the predicted value) (4). Predicted FEV1 values were calculated using the Global Lung Function Initiative (GLI-2012) reference equations, implemented via specialized software available at (https://gli-calculator.ersnet.org/index.html) (19).

### Covariates

Demographic data (age, gender, race/ethnicity, and poverty income ratio), health-related behaviors (smoking status and alcohol consumption), medical history (cardiovascular disease and stroke) were collected from NHANES through standardized questionnaires. Ethnicity was categorized as Mexican American, other Hispanic, non-Hispanic White, non-Hispanic Black, and other races. The poverty income ratio (PIR) was calculated as the ratio of monthly family income to the federal poverty level, following the Department of Health and Human Services guidelines, and categorized into low income (≤1.30), middle income (1.31–3.50), and high income (>3.50) (20). Body mass index (BMI) was categorized into normal weight (<25 kg/m²), overweight (25–29.9 kg/m²), and obese (≥30 kg/m²), and included as a categorical variable in multivariable regression analyses. Cardiovascular disease and stroke were identified based on affirmative responses to the following question: “Has a doctor or other health professional ever told you that you had congestive heart failure, coronary heart disease, angina, heart attack, or stroke?” Alcohol consumption was determined by asking, “Have you had at least 12 alcoholic drinks in the past year?” Smoking status was defined as a binary variable (yes/no), based on responses to the questions: “Have you smoked at least 100 cigarettes in your lifetime?” and “Do you currently smoke?” Additionally, laboratory data included cotinine, alanine aminotransferase (ALT), aspartate aminotransferase (AST), and creatinine. Renal function was assessed by calculating the estimated glomerular filtration rate (eGFR) using the CKD-EPI equation (21), and eGFR was included as a continuous covariate in the models.

### Statistical analysis

We conducted weighted analyses according to NHANES guidelines. Continuous variables that did not follow a normal distribution were expressed as medians with interquartile ranges, and group comparisons were performed using the Mann-Whitney U test. Categorical data were presented as proportions, with group comparisons using the chi-square test. Ordinal data were also expressed as proportions, with group comparisons performed using the Mann-Whitney U test. To examine the association between eGDR and PRISm, we employed weighted multivariable logistic regression, constructing three models: Model 1: Unadjusted; Model 2: Adjusted for gender, age, ethnicity, and PIR; Model 3: Adjusted for all covariates (gender, age, ethnicity, PIR, BMI, cotinine, AST, ALT, GFR creatinine clearance, cardiovascular disease, stroke, alcohol consumption, and smoking status). We also conducted subgroup and interaction analyses to explore the relationship between eGDR and PRISm across different populations. To assess potential nonlinear associations, we used restricted cubic spline analysis. Finally, we performed a receiver operating characteristic (ROC) analysis to evaluate the predictive ability of eGDR for PRISm. All statistical analyses were performed using R software (version 4.0.0) and SPSS (version 25.0), with statistical significance set at *P* < 0.05.

## Results

### Baseline characteristics

In **Table 1**, a total of 13,661 participants were included in the final analysis after excluding those with missing data on PRISm (n = 10,954) and eGDR (n = 5,827). The median eGDR for the overall sample was 11.89 (IQR: 9.08–13.33). Participants in the PRISm group had significantly lower median eGDR values compared to the non-PRISm group [9.92 (IQR: 8.04–12.49) vs. 12.01 (IQR: 9.22–13.38); *P* < 0.001], indicating greater insulin resistance in the PRISm group. The PRISm group was older, with a median age of 48 (IQR: 34–61) compared to 44 (IQR: 30–59) in the non-PRISm group (P < 0.001). Cotinine levels were also higher in the PRISm group [0.09 (IQR: 0.02–30.55) vs. 0.05 (IQR: 0.02–12.70); *P* < 0.001] and median eGFR was significantly lower in the PRISm group [85.47 (IQR: 64.70–103.07) vs. 89.84 (IQR: 69.66–108.48); *P* < 0.001]. No significant differences were observed in ALT (P = 0.332) and AST (P = 0.167) levels between the two groups.

**Table 1:** Baseline characteristics of the study population.

Variables	Total (n = 13661)	Non-PRISm (n = 12493)	PRISm (n = 1168)	Statistic	*P*
eGDR (mg/kg/min), M (Q_1_, Q_3_)	11.89 (9.08, 13.33)	12.01 (9.22, 13.38)	9.92 (8.04, 12.49)	-14.29	<0.001
Quantile, n (%)				-14.24	<0.001
Q1	3412 (24.98)	2928 (23.44)	484 (41.44)		
Q2	3418 (25.02)	3108 (24.88)	310 (26.54)		
Q3	3415 (25.00)	3215 (25.73)	200 (17.12)		
Q4	3416 (25.01)	3242 (25.95)	174 (14.90)		
Age, M (Q_1_, Q_3_)	44.00 (30.00, 59.00)	44.00 (30.00, 59.00)	48.00 (34.00, 61.00)	-5.20	<0.001
Sex, n (%)				0.00	0.965
Male	6991 (51.17)	6394 (51.18)	597 (51.11)		
Female	6670 (48.83)	6099 (48.82)	571 (48.89)		
Ethnicity, n (%)				853.73	<0.001
Mexican American	2289 (16.76)	2206 (17.66)	83 (7.11)		
Other Hispanic	1491 (10.91)	1413 (11.31)	78 (6.68)		
Non-Hispanic White	5713 (41.82)	5471 (43.79)	242 (20.72)		
Non-Hispanic Black	2957 (21.65)	2327 (18.63)	630 (53.94)		
Other Race	1211 (8.86)	1076 (8.61)	135 (11.56)		
PIR, n (%)				-3.38	<0.001
≤1.3	4144 (33.08)	3772 (32.86)	372 (35.50)		
>1.3 and ≤3.5	4510 (36.01)	4099 (35.71)	411 (39.22)		
>3.5	3872 (30.91)	3607 (31.43)	265 (25.29)		
BMI (kg/m^2^), n (%)				-10.39	<0.001
<25	4439 (32.53)	4159 (33.32)	280 (24.08)		
≥25 and < 30	4438 (32.52)	4140 (33.17)	298 (25.62)		
<30	4768 (34.94)	4183 (33.51)	585 (50.30)		
Cotinine (ng/mL), M (Q_1_, Q_3_)	0.05 (0.02, 13.25)	0.05 (0.02, 12.70)	0.09 (0.02, 30.55)	-5.08	<0.001
eGFR (mL/min/1.73 m²), M (Q_1_, Q_3_)	89.41 (69.17, 108.08)	89.84 (69.66, 108.48)	85.47 (64.70, 103.07)	-6.06	<0.001
ALT (U/L), M (Q_1_, Q_3_)	21.00 (16.00, 28.00)	21.00 (16.00, 28.00)	21.00 (16.00, 28.00)	-0.97	0.332
AST (U/L), M (Q_1_, Q_3_)	23.00 (20.00, 28.00)	23.00 (20.00, 28.00)	23.00 (19.00, 28.00)	-1.38	0.167
Smoke, n (%)				0.00	0.978
Yes	5525 (45.36)	5031 (45.37)	494 (45.32)		
No	6655 (54.64)	6059 (54.63)	596 (54.68)		
Alcohol, n (%)				68.28	<0.001
Yes	8725 (74.60)	8062 (75.64)	663 (63.93)		
No	2971 (25.40)	2597 (24.36)	374 (36.07)		
Heart Disease, n (%)				65.31	<0.001
Yes	685 (5.62)	565 (5.09)	120 (11.00)		
No	11500 (94.38)	10529 (94.91)	971 (89.00)		
Stroke, n (%)				22.41	<0.001
Yes	261 (2.14)	216 (1.95)	45 (4.12)		
No	11913 (97.86)	10867 (98.05)	1046 (95.88)		

eGDR, Estimated Glucose Disposal Rate; PRISm, Preserved Ratio Impaired Spirometry; BMI: Body Mass Index; eGFR, Estimated Glomerular Filtration Rate; ALT, Alanine Aminotransferase; AST, Aspartate Aminotransferase.

Ethnicity was significantly associated with PRISm status (*P* < 0.001). PRISm was most prevalent in non-Hispanic Black participants (53.94%), and least common in Mexican American (7.11%). BMI was also significantly associated with PRISm (*P* < 0.001), with a higher proportion of obese individuals in the PRISm group (50.30%) compared to the non-PRISm group (33.51%). Smoking status did not differ significantly between the two groups (*P* = 0.978). However, alcohol consumption was significantly lower in the PRISm group, with only 63.93% reporting alcohol consumption compared to 75.64% in the non-PRISm group (*P* < 0.001). Cardiovascular disease and stroke were more common in the PRISm group, with heart disease present in 11.00% of PRISm cases compared to 5.09% in the non-PRISm group (*P* < 0.001), and stroke present in 4.12% of PRISm cases compared to 1.95% in the non-PRISm group (*P* < 0.001).

### Logistic regression models

Weighted multivariable logistic regression analysis demonstrated a significant negative association between eGDR and PRISm (**Table 2**). In the unadjusted model (Model 1), each unit increase in eGDR was associated with a 15.1% reduction in the odds of PRISm (OR = 0.849, 95% CI: 0.820–0.880; *P* < 0.001). After adjusting for gender, age, race/ethnicity, and poverty income ratio (Model 2), the association remained significant (OR = 0.849, 95% CI: 0.818–0.881; *P* < 0.001). In the fully adjusted model (Model 3), which included additional covariates such as BMI, cotinine, ALT, AST, GFR, cardiovascular disease, stroke, alcohol consumption, and smoking, each unit increase in eGDR was associated with a 7.3% reduction in the odds of PRISm (OR = 0.927, 95% CI: 0.880–0.976; *P* = 0.005). When eGDR was categorized into quartiles, the highest quartile (Q4) was associated with a 41.7% lower risk of PRISm compared to the lowest quartile (Q1) in the fully adjusted model (OR = 0.583, 95% CI: 0.393–0.867; *P* = 0.009). A significant trend was observed across quartiles (*P* for trend = 0.002), further supporting a negative relationship between eGDR and PRISm.

**Table 2:** Multivariate logistic regression analysis of the association between eGDR and PRISm across different models.

Variables	Model 1		Model 2		Model 3	
	*OR* (95% CI)	*P*	*OR* (95% CI)	*P*	*OR* (95% CI)	*P*
eGDR	0.849 (0.820, 0.880)	<0.001	0.849 (0.818, 0.881)	<0.001	0.927 (0.880, 0.976)	0.005
Categories						
Q 1	Reference	/	Reference	/	Reference	/
Q 2	0.611 (0.484, 0.772)	<0.001	0.620 (0.470, 0.817)	0.001	0.785 (0.585, 1.055)	0.106
Q 3	0.405 (0.316, 0.520)	<0.001	0.441 (0.350, 0.556)	<0.001	0.660 (0.501, 0.869)	0.004
Q 4	0.362 (0.281, 0.467)	<0.001	0.310 (0.232, 0.415)	<0.001	0.583 (0.393, 0.867)	0.009
*P* for trend	/	<0.001	/	<0.001	/	0.002

Model 1: Unadjusted; Model 2: Adjusted for gender, age, ethnicity, poverty income ratio; Model 3: Adjusted for all covariates (gender, age, ethnicity, PIR, BMI, cotinine, AST, ALT, GFR creatinine clearance, cardiovascular disease, stroke, alcohol consumption, and smoking status); eGDR, Estimated Glucose Disposal Rate; PRISm, Preserved Ratio Impaired Spirometry; OR, Odds ratio; CI, Confidence Interval.

### Subgroup and interaction analysis

Subgroup analyses revealed significant differences in the relationship between eGDR and PRISm across gender and age groups (**Table 3**). Among women, eGDR was significantly associated with lower odds of PRISm (OR = 0.874, 95% CI: 0.821–0.929; *P* < 0.001), while no significant association was observed in men (*P* = 0.702). The interaction between gender and eGDR was significant (*P* = 0.012), indicating that the association was stronger in women. Similarly, a significant association was found in participants over 40 years of age (OR = 0.913, 95% CI: 0.848–0.982; *P* = 0.016), but not in those aged 40 or younger (*P* = 0.146), with a significant interaction effect for age (*P* = 0.016). No significant interactions were observed between eGDR and race (*P* = 0.408) or poverty income ratio (*P* = 0.984), although significant associations between eGDR and PRISm were found in Mexican American, Non-Hispanic Black ​racial groups.

**Table 3:** Subgroup and interaction analysis of eGDR and PRISm by gender, age, ethnicity, and poverty ratio.

Subgroup	*OR* (95% CI)	*P*	*P* for interaction
Overall	0.927 (0.880, 0.976)	0.005	
Gender			0.012
Male	0.938 (0.669, 1.315)	0.702	
Female	0.874 (0.821, 0.929)	<0.001	
Age			0.016
≤40	0.949 (0.883, 1.019)	0.146	
>40	0.913 (0.848, 0.982)	0.016	
Ethnicity			0.408
Mexican American	0.732 (0.646, 0.83)	<0.001	
Other Hispanic	0.860 (0.715, 1.033)	0.104	
Non-Hispanic White	0.927 (0.857, 1.002)	0.055	
Non-Hispanic Black	0.908 (0.858, 0.961)	0.002	
Other Race	1.073 (0.896, 1.285)	0.433	
PIR			0.984
≤1.3	0.941 (0.873, 1.014)	0.109	
>1.3 and ≤3.5	0.912 (0.846, 0.983)	0.017	
>3.5	0.939 (0.854, 1.034)	0.193	

eGDR, Estimated Glucose Disposal Rate; PRISm, Preserved Ratio Impaired Spirometry; OR, Odds ratio; CI, Confidence Interval.

### Nonlinear and ROC analysis

**Figure 2:**
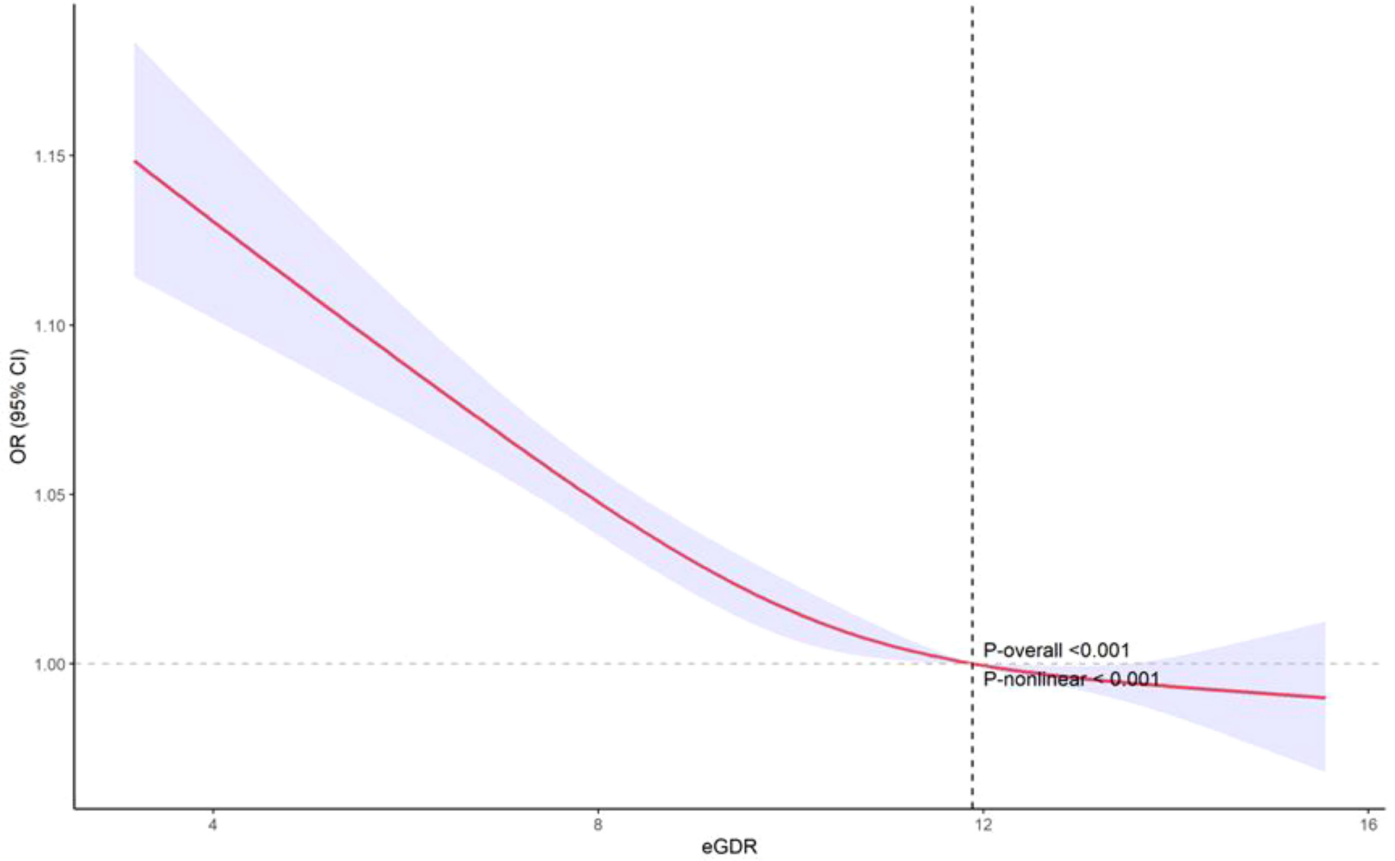
Nonlinear relationship between eGDR and PRISm: restricted cubic spline analysis. eGDR, Estimated Glucose Disposal Rate; PRISm, Preserved Ratio Impaired Spirometry.

**Figure 3:**
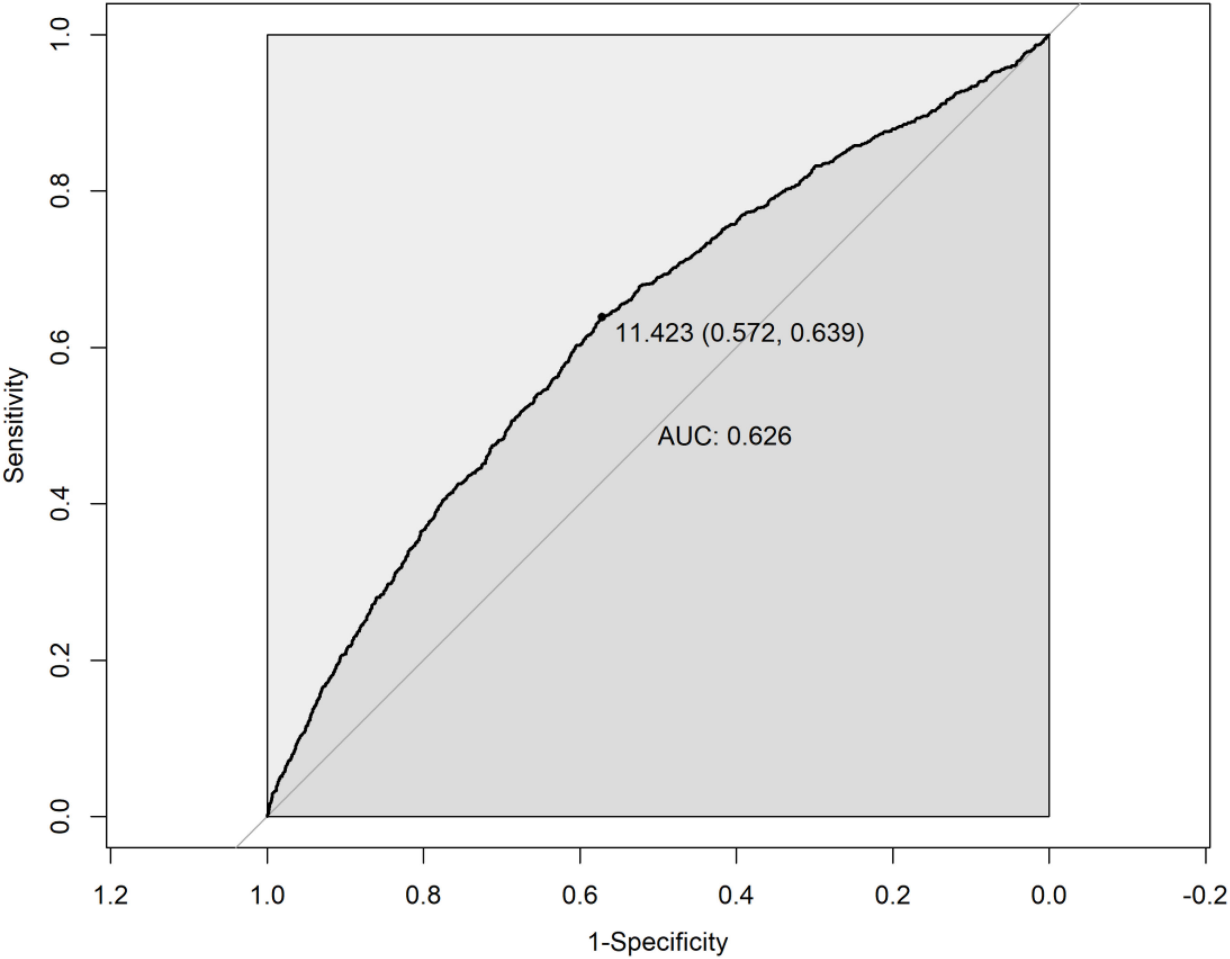
Receiver operating characteristic (ROC) curve for eGDR in predicting PRISm. eGDR, Estimated Glucose Disposal Rate; PRISm, Preserved Ratio Impaired Spirometry.

The restricted cubic spline (RCS) analysis revealed a significant nonlinear relationship between eGDR and PRISm (*P*-nonlinear < 0.001). As shown in **Figure 2**, the OR for PRISm decreases as eGDR increases, with the most pronounced reduction occurring at lower eGDR levels. Beyond an eGDR value of approximately 12 mg/kg/min, the association stabilizes, with the OR approaching 1. This indicates that higher eGDR levels are associated with a lower likelihood of PRISm, but the effect diminishes as eGDR increases. ROC analysis revealed that the area under the curve (AUC) for eGDR predicting PRISm was 0.626, indicating modest predictive accuracy (**Figure 3**). The optimal cutoff value for eGDR was 11.423 mg/kg/min, with a sensitivity of 63.9% and specificity of 57.2%.

## Discussion

Preserved ratio impaired spirometry (PRISm) is a newly defined phenotype of lung function impairment, characterized by individuals exhibiting a normal FEV1/FVC ratio, while having an FEV1 less than 0.8 times the predicted value (5). Although PRISm shares some features with both obstructive and restrictive lung patterns, it is distinct in that it does not follow the typical patterns of either (10). PRISm is associated with various adverse clinical outcomes, such as increased respiratory symptoms, elevated comorbidity rates of hypertension and diabetes, and higher mortality rates (4, 7, 8). Furthermore, PRISm is a heterogeneous condition, with only a subset of individuals progressing to COPD, suggesting that PRISm may represent a unique clinical phenotype with its own pathophysiological and prognostic implications (6, 9). This study investigates the relationship between estimated glucose disposal rate (eGDR), an indicator of insulin resistance, and PRISm. Our results show that lower eGDR is significantly associated with an increased risk of PRISm, suggesting a negative relationship between insulin resistance and PRISm.

The baseline characteristics of participants further clarified the differences between the PRISm and non-PRISm groups. Notably, the PRISm group was older, had higher cotinine levels, lower eGFR, and a higher prevalence of cardiovascular diseases and obesity. While it is widely acknowledged that smoking impairs lung function, cotinine is specifically associated with reduced lung function and airflow obstruction (22). Additionally, Obesity, a recognized risk factor for both insulin resistance and respiratory diseases, increases airway resistance while simultaneously altering breathing patterns, thereby affecting ventilation and oxygenation (23); at the same time, the accumulation of visceral adipose tissue due to obesity is closely associated with a higher incidence of respiratory diseases (24). However, in the multivariable logistic regression analysis, eGDR remained significantly negatively correlated with PRISm even after fully adjusting for confounding factors such as sex, age, race, BMI, cotinine levels, liver and kidney function, cardiovascular disease, smoking, and alcohol consumption. This suggests that higher insulin sensitivity, as reflected by higher eGDR, is associated with a lower risk of developing PRISm, independent of these potential confounders.

In the multivariable logistic regression analysis, all models demonstrated a significant negative association between eGDR and PRISm. In both unadjusted and adjusted models, each unit increase in eGDR was associated with a 15.1% reduction in the likelihood of PRISm. This relationship persisted in the fully adjusted model, where even after accounting for potential confounders such as sex, age, ethnicity, BMI, cotinine levels, renal and hepatic function, cardiovascular disease, smoking, and alcohol consumption, each unit increase in eGDR was still linked to a 7.3% decrease in the likelihood of PRISm.

These findings suggest that higher eGDR levels, indicative of better insulin sensitivity, are associated with a lower risk of PRISm. This relationship may be partly explained by the characteristics of participants with moderate or severe insulin resistance, who often present with systemic inflammation—marked by elevated levels of white blood cells, neutrophils, and plasma interleukin-6—and dyslipidemia, characterized by high triglycerides and low HDL cholesterol. Insulin resistance is typically accompanied by chronic low-grade inflammation, which promotes the release of inflammatory mediators such as tumor necrosis factor-α and interleukin-6 (25, 26). These factors not only impair systemic metabolism but also directly affect lung tissue, leading to airway inflammation and structural remodeling, which contribute to airflow limitation and reduced lung function (27–29). Additionally, insulin resistance increases oxidative stress in the body, which refers to an imbalance between the production of free radicals and antioxidant defenses. Elevated levels of free radicals can damage cells, including lung cells, causing dysfunction and structural damage (30). This damage not only compromises airway patency but may also trigger an inflammatory response in the lungs, further exacerbating lung function impairment (31). Beyond systemic effects, insulin resistance may impair lung function via adipose tissue dysfunction, which promotes pro-inflammatory adipokines like resistin and reduces anti-inflammatory adiponectin (32, 33). Resistin is linked to asthma, COPD, fibrosis, and acute lung injury, while adiponectin suppresses pulmonary inflammation by inhibiting TNF-α, IL-6, and chemokine production (34, 35). These adipokine shifts may mediate the adverse impact of insulin resistance on lung health. Moreover, chronic hyperinsulinemia may also interfere with cellular repair and regeneration pathways in the lung, limiting the ability to recover from environmental or inflammatory insults (36).

Subgroup analyses in our study revealed that this association was more pronounced among females and individuals aged over 40 years. The stronger association in women may be attributable to differences in body fat distribution and hormonal regulation (37, 38). Sex-specific patterns in insulin resistance, influenced by sex steroid hormones, may partly explain this finding. Estrogens play a protective role in metabolic regulation, and their decline after menopause contributes to increased insulin resistance and diabetes risk (39). A Evidence from both human genetics and animal models has shown that disruption of estrogen signaling—such as through aromatase or estrogen receptor α deficiency—can lead to marked metabolic dysfunction (38). Moreover, estrogen, which has anti-inflammatory effects, may play a protective role in premenopausal women; with age-related hormonal changes, metabolic dysregulation may have a more deleterious impact on lung function (40). These findings suggest that insulin resistance may have a more pronounced impact on lung function in women due to hormone-related differences in insulin sensitivity and inflammation. In older adults, age-related skeletal muscle dysfunction—characterized by mitochondrial impairment, metabolic dysregulation, inflammation, and sarcopenia—leads to reduced insulin sensitivity and is a key mechanism underlying insulin resistance in the elderly (41). The synergistic effects of sarcopenia and insulin resistance can exacerbate systemic inflammation and oxidative stress, both of which are known to impair lung function (14, 42). Additionally, age-related declines in lung elasticity, respiratory muscle strength, and ventilatory responsiveness may render older adults more vulnerable to the adverse effects of metabolic abnormalities on pulmonary function (43). In contrast, no significant interaction was observed between eGDR and race or poverty-to-income ratio; however, notable associations were found within specific racial groups. Notably, significant associations between eGDR and PRISm were observed in Non-Hispanic Black and Mexican American participants. This finding aligns with prior studies indicating that both racial groups exhibit higher levels of insulin resistance and insulin secretion compared to non-Hispanic Whites. For instance, Haffner et al. reported that both Non-Hispanic Black and Mexican American individuals showed significantly higher levels of insulin resistance than their non-Hispanic White counterparts (44). Similarly, Hasson et al. highlighted the heightened insulin resistance and upregulated beta-cell function in African Americans, potentially contributing to their elevated risk of metabolic diseases (45). These metabolic characteristics may also influence pulmonary outcomes, thereby partially explaining the higher prevalence of PRISm in these populations.

The non-linear relationship observed in the restricted cubic spline analysis indicates that while higher levels of eGDR are associated with a decreased likelihood of PRISm, this association tends to plateau once eGDR exceeds approximately 12 mg/kg/min. This suggests a potential threshold effect, beyond which further improvements in insulin sensitivity confer minimal additional benefit in reducing PRISm risk. Such a plateau is biologically plausible, as metabolic improvements may only translate to clinical benefits up to a certain point, after which risk stabilizes. Interventions targeting insulin resistance may therefore be particularly beneficial for individuals with lower baseline eGDR. Additionally, ROC curve analysis demonstrated that eGDR has a certain predictive accuracy in identifying PRISm, with an AUC of 0.626. Although the AUC indicates only limited discriminatory power, this result suggests that eGDR may be more suitable as a metabolic health risk indicator rather than a standalone diagnostic tool for PRISm. In future research or clinical practice, eGDR could be combined with other biomarkers—such as inflammatory markers, lung imaging parameters, or genetic risk scores—to enhance predictive performance and facilitate early identification of high-risk individuals.

Overall, our findings demonstrate that lower eGDR, indicating higher insulin resistance, is significantly associated with increased PRISm risk, independent of common confounders. This association is stronger in women and older adults, likely due to hormonal and age-related physiological changes. The observed threshold effect suggests that improving insulin sensitivity may be most beneficial in individuals with lower baseline eGDR. While eGDR alone has limited predictive power, it may serve as a useful metabolic marker when combined with other indicators to better identify individuals at risk for PRISm.

### Limitations

This study has several limitations that should be acknowledged. First, the cross-sectional design restricts our ability to establish causal relationships between eGDR and PRISm, as we can only infer associations rather than direct causation. Additionally, the reliance on self-reported data for lifestyle factors, such as smoking and alcohol consumption, may introduce bias or inaccuracies. The use of eGDR as a surrogate measure of insulin sensitivity, while clinically relevant, may not capture all aspects of metabolic health, potentially leading to residual confounding. Furthermore, although we adjusted for numerous known confounders, residual confounding from unmeasured or unknown variables—such as environmental exposures, detailed dietary patterns, genetic predispositions, and undiagnosed comorbidities—cannot be completely ruled out. Moreover, the generalizability of our findings may be limited, as the study population primarily consisted of adults from specific demographic groups, which may not fully represent the broader population. Finally, while we adjusted for several potential confounders, residual or unrecognized confounders may still influence the observed associations. Future research should aim to address these limitations through longitudinal designs and more comprehensive assessments of metabolic health and environmental factors.

## Conclusion

In conclusion, our study demonstrates a significant inverse association between estimated eGDR and PRISm among a diverse population of US adults. Participants with lower eGDR values exhibited a higher prevalence of PRISm, indicating greater insulin resistance and potential metabolic dysfunction. The findings suggest that eGDR may serve as a valuable marker for assessing the risk of PRISm, particularly among women and older adults. Given the growing recognition of the interplay between metabolic health and respiratory function, further research is warranted to elucidate the underlying mechanisms linking insulin resistance and pulmonary impairment.

## Data Availability

The original contributions presented in the study are included in the article/**Supplementary Material**. Further inquiries can be directed to the corresponding author.
